# The Threat of Ambient Air Pollution in Kathmandu, Nepal

**DOI:** 10.1155/2018/1504591

**Published:** 2018-07-10

**Authors:** Bhuvan Saud, Govinda Paudel

**Affiliations:** Department of Medical Laboratory Technology, Janamaitri Foundation Institute of Health Sciences (JFIHS), GPO Box 8322, Hattiban, Lalitpur, Nepal

## Abstract

Air pollution has been a major problem of 21st century for both developed and developing world. It has a negative impact on various environmental aspects which directly or indirectly affect the quality of human health. Nepal, especially Kathmandu, in the current situation, is observing rapid urbanization and various infrastructure development projects. As a result, these sorts of human activities have been responsible for increasing air pollution in an enormous rate inside Kathmandu Valley. Chronic exposure of deteriorated air increases the chance of Noncommunicable Disease (NCD) like lung disease, heart disease, and cancers. Short term exposures also invite respiratory diseases and allergy. This review is an attempt to summarize the updated knowledge on the threat of air pollution on public health and discuss the sources of air pollutants in Kathmandu. We reviewed the literatures that were published in PMC, MEDLINE, life science journals, and organization official websites and finally came up with the findings and their interpretation that reveal the current scenario in the context of Kathmandu's air quality status and its impact on human health. The knowledge about the invisible killer's role in causing acute and chronic diseases may help in finding out the answer of the question regarding its effect and prevention.

## 1. Introduction

Kathmandu Valley, well known as city of temples, has now transformed itself into city of pollution. The city of temples is now clad in dust and smoke. The pristine blue hills and the crisp blue sky that covered the valley just about two decades ago now appear gray and hazy due to the stagnant smog that hovers over them. Kathmandu has a population density of 13,225 per km^2^ [1] as of data recorded by Central Bureau of Statistics in 2011, with population growth rate of 4.78% [2]. Such a high population in the valley is due to its being the capital city and people from all over the country throng to the city in pursuit of better life and opportunities. The valley is surrounded by high mountains ranging from 2000 to 2800 metres from sea level [3]. Due to this, the valley has a unique bowl-shaped topographic structure which restricts the movement of wind thereby retaining the pollutants in the air [4–6]. This makes the valley particularly vulnerable to air pollution.

World Health Organization (WHO) defines air pollution as contamination of the indoor or outdoor environment by any chemical, physical, or biological agent that modifies the natural characteristics of the atmosphere. Common sources of air pollution are household combustion devices, motor vehicles, industrial facilities, and forest fires [7]. Air pollution is a complex mixture of thousands of components, majority of which include airborne Particulate Matter (PM) and gaseous pollutants like ozone (O3), nitrogen dioxide (NO2), volatile organic compounds (like benzene), carbon monoxide (CO), sulfur dioxide (SO2), etc. [8, 9]. A variety of respiratory and other diseases, which can also be fatal, are caused by outdoor and indoor air pollution [7]. Particulate Matter (PM 10) is that suspended particle that is about 10*µ*m in diameter and mainly arises from the poor quality roads, construction sites, and farms and is responsible for causing irritation in eyes, nose, and acute respiratory infections [10]. High rate of PM10 associated mortality and respiratory illness are found in children and adults [11]. On the other hand PM2.5 (particles less than 2.5 *µ*m in diameter) penetrate deep into the lung, irritate and corrode the alveolar wall, consequently impair lung function [12], and even penetrate the blood [7]. It has been shown that PM2.5 is a public health concern whose exposure leads to decreased life expectancy [13–16]. The high concentration of CO forms carboxyhemoglobin (COHb) and exacerbates heart attack and also affects nervous system, NO2 causes bronchitis and bronchopneumonia, and SO2 causes eye irritation, shortness of breath, chronic bronchitis, asthma, various heart diseases, lung disease, cancer [11], and conjunctivitis [17]. O3 is associated with stimulation of transcription factors and increased expression of cytokine and adhesion molecules which lead to the development of cardiovascular and respiratory diseases [18–20]. Air pollution's association with autoimmune diseases has been published [21, 22]. Air pollution has been emerging as a major threat to the whole ecosystem.

Geographically, Nepal is a small landlocked country sandwiched between two giant countries India and China. Though these two countries have been emerging as supreme economic powers, they still struggle in managing their environmental air quality. Studies have shown that major cities of India like Delhi, Raipur, Gwalior, and Lucknow are listed among the world's top 10 polluted cities and altogether 37 Indian cities feature in a list of 100 most polluted cities globally, with highest PM10 [25]. Delhi the capital of India is classed as the world's most polluted capital city with air pollution parameters 30 times higher than WHO's recommended upper limit [26]. China a rapidly developing country equally suffers from air pollution. Rapid industrialization and high energy consumption have been the major reasons of air pollution in China. Cities such as Jingjinji, Beijing, Tianjin, and Chongjin and northwest part are the places that are highly polluted [27]. PM2.5 is considered the main pollutant of atmospheric pollution in China [28]. It was found that average PM2.5 concentration among 210 cities in China is approximately more than 8 times higher than WHO recommended level [29]. In Nepal, along with the rapid and uncontrolled urbanization and haphazard developmental projects, people are being victimized with serious airborne diseases. Though few studies and publications have been done regarding air pollution in Kathmandu, the city has now been regarded as severely polluted place [30]. This article hence emphasizes highlighting the effects, sources, status, and threats of air pollution in Kathmandu Valley.

## 2. Sources

**Figure 1:**
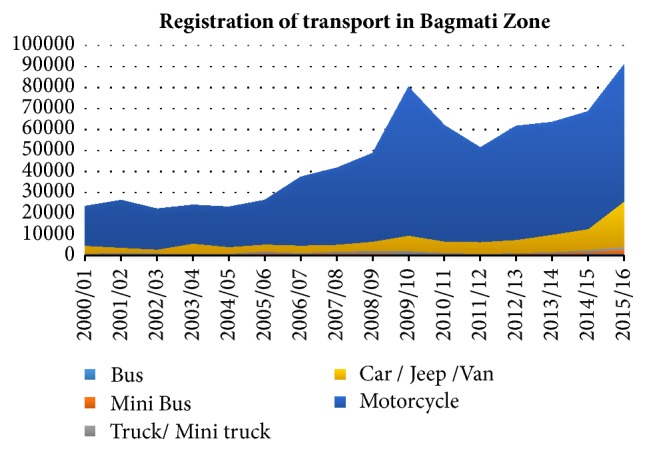
Registration of vehicles in Bagmati Zone 2001 to 2016 [23].

A variety of factors are responsible for deteriorating the quality of air. Nepal is a rapidly urbanizing country. A data of 2014 shows 4.6 million of Nepalese live in urban areas [31]. This trend is increasing rapidly and it is estimated that urban population will reach 60 million by 2040 [32]. Subsequent increase in number of vehicles is one of the main culprits of air pollution [33, 34]. Kathmandu Valley has seen a rapid increase in vehicle numbers in the last 15 years. Data have shown that in 2000/1, number of registered vehicles was 24,003 and by 2015/16 it has increased to 7, 79,822. This shows an increment by more than 32 times in the last one and a half decade. The graph shown in Figure 1 illustrates the vehicles registered on different categories among which private vehicles like motorcycles and cars top the list, respectively. The trend of purchasing new vehicle is also seen to be increasing as the year 2015/16 sees the largest number of registered vehicles [23]. Private vehicles are increasing in comparison to public transport vehicle. Due to lack of an efficient public transport system, many residents have chosen to buy private vehicle. Emissions from vehicles are particularly toxic as diesel powered vehicles, which are considered deadly pollutant and carcinogen, are more numerous than the petrol powered ones. This fact agrees with the report of WHO where it has stated that low and middle income countries suffer superfluously from transport generated pollution due to old and inefficient diesel powered vehicles [35]. Besides vehicles, haphazard digging of road for currently ongoing Melamchi water project, brick kilns, unplanned expansion of roads, ill-managed dumping of building materials on the busy road sides, and the old engine vehicles that race incessantly on the pothole laden roads are adding insult to injury.

## 3. Status

**Figure 2:**
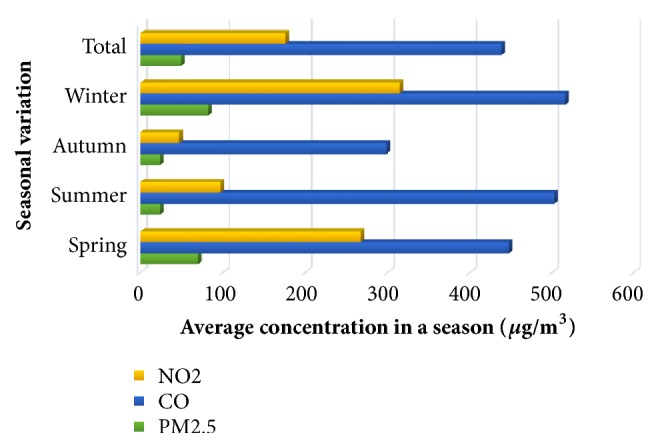
Outdoor air pollution in Kathmandu Valley, Nepal, 2015 [24].

In 2016, Environmental Performance Index (EPI) of Nepal's air quality ranked 177th out of 180 countries [36] and, in Asia, Kathmandu is ranked one of the most polluted cities [4]. According to a report of World Health Organization (WHO), the maximum status of fine Particulate Matter (PM2.5) in urban areas of Nepal was noted to be 140 *µ*g/m^3^ [37] which is 10 times higher than the desirable value. Ministry of Science and Technology, in 2012, had published a guideline on “National Ambient Air Quality.” The values set on these parameters were still higher than those set by WHO [38]. The targeted PM10 and PM2.5 values were 120 *µ*g/m^3^ and 40 *µ*g/m^3^, respectively, which were approximately two times higher than the WHO targeted value. The Department of Environment is planning to establish air quality monitoring stations throughout the country. As of today, it is limited in nine places, 3 stations inside Kathmandu Valley and 6 stations outside the valley [39]. According to Department of Environment, in 2017, 24-hour average of Total Suspended Particles (TSP) in a site in Kathmandu was 4,749 *µ*g/m^3^, average PM10 was 2,928 *µ*g/m^3^, and PM 2.5 was 226 *µ*g/m^3^ [40].To find out seasonal variation of air pollution, a study conducted in Kathmandu Valley measured NO2, CO, and PM 2.5 concentration on daily basis in all the four seasons of a year. The maximum level of each of these parameters was seen during winter and spring seasons as shown in Figure 2 [24].

## 4. Impact on Human Health

Till date, majority of studies on impacts of air pollution on human health have been done in North America and Europe. Only few studies on this regard have been done in region like Nepal. It has been found that, with high level of air pollution exposure, Nepal, especially Kathmandu, is suffering from a potentially serious human health burden from air pollution [30]. Air pollution has long been regarded as a silent killer responsible for causing a variety of chronic and infectious diseases. Globally, unhealthy environment causes a total of 12.6 million deaths [41] and air pollution is solely responsible for 7 million deaths annually [42]. Every year air pollution is linked with around 6.5 million premature deaths globally, of which household air pollution causes 3.5 million and ambient air pollution causes 3 million deaths and the future scenario by the 2040 is assumed to increase up to 7.5 million premature deaths per year [43]. PM2·5 caused an estimated 7·6% of total global mortality in 2015 and was the fifth-ranking global mortality risk factor. Although global rates of mortality due to PM2·5 exposure decreased from 1990 to 2015 as a result of improved air quality in high-income countries [44], in Nepal's case the impact on human health is equally severe. By 2030 annual premature deaths in Nepal, due to outdoor air pollution, are expected to be 24,000 [45].

In Nepal, the commonest diseases like respiratory illness, allergy, and eye infection and chronic diseases like lung cancer, chronic obstructive pulmonary disease (COPD), Ischaemic Heart Disease (IHD), and stroke are present in an alarming rate. Noncommunicable Diseases (NCDs) have been the major cause of human death accounting for 60% of deaths. Of NCDs cardiovascular diseases have caused a maximum death of 22% followed by chronic respiratory diseases 13%, cancer 8%, and other NCDs 14%. Premature (between ages of 30 and 70 years) mortality due to NCDs is 22% [31]. Data of Global Health Observatory (GHO) for mortality from ambient air pollution in Nepal in 2012 shows a threatening rate of 9,944 deaths of which Ischaemic Heart Disease (IHD) caused the highest death rate of 33.4% followed by stroke 32%, chronic obstructive pulmonary disease (COPD) 17.8%, lung cancer 9.3%, and Acute Lower Respiratory Tract Infection (ALRTI) 7.4%. The number of female deaths was higher than that of male deaths in each disease [37]. Data of Department of Health (DoH) services in Nepal shows that, in 2013-2014, COPD was the most common cause of mortality among inpatients and respiratory tract diseases were the most common reason for outpatients' consultations with both upper and lower respiratory tract infections being within the top four [46].

**Figure 3:**
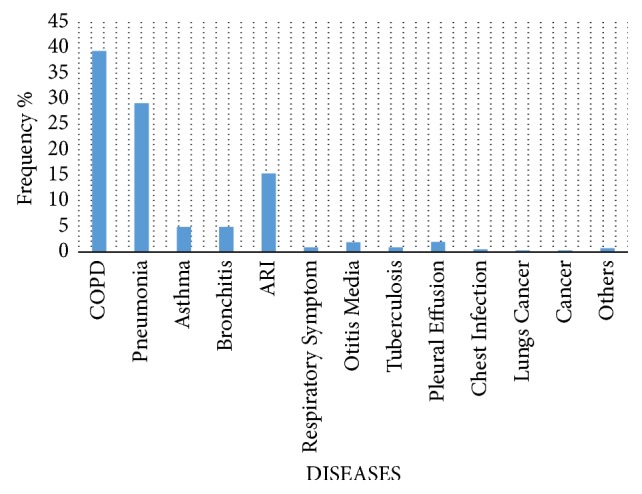
Disease-wise distribution of hospitalized patient in Kathmandu Valley [24].

In a hospital based study, NCD prevalence was 31% out of which COPD was 43%, cardiovascular disease was 40%, and cancer was 5% [47]. A study done in hospitalized patients of various hospitals of Kathmandu Valley shows a high prevalence of respiratory diseases. Among the diseases, COPD was the most prevalent one with a significant proportion of other diseases too as shown in Figure 3. Gender-wise distribution showed that of total inpatients 51.3% were male and 48.7% were female. District-wise distribution showed that highest number of patients came from Kathmandu 44.4% followed by Lalitpur 10.3% and Bhaktpur 10.2%. Overall morbidity was 44.4% and the highest morbidity rate was seen in COPD cases as shown in Figure 3 [24]. A hospital based study outside Kathmandu Valley in Chitwan showed that 48.4% of COPD patients had a history of the disease since more than 5 years [48]. A study in 2017 has shown that 2.7–3.4 million preterm births might be associated with PM2.5 exposure in 2010 globally [49]. Nepal also has a significant number of preterm births of 14% [50]. No clear cut reasons have been found for this high preterm birth, but air pollution can arguably have an important impact on this. Air pollution is rising as an occupational hazard in Nepal, both in Kathmandu and in other cities like Pokhara, especially in traffic police who are being continuously exposed to dusty roads [51, 52]. Due to this pulmonary functions have been significantly worsened in the traffic police working in Kathmandu [53]. Airborne occupational hazards are equally present in brick kiln workers and grocery workers in Kathmandu, whose health has been seriously hampered and needs quick action for protection [54, 55]. According to World Bank, air pollution stands as fourth major factor for causing death worldwide leaving metabolic risks, dietary risks, and tobacco smoking behind. Globally, 1 in 10 deaths is understood to be caused by air pollution [56]. Hazards due to air pollution have been a great economic burden too. A data on impact of air pollution on human health shows a global loss of $225 billion annually of which South Asia has been the most severely affected region suffering a loss of more than $66 billion alone annually, which is approximately 1% of the Gross Domestic Product (GDP) [57].

## 5. Legislatives and Future Action Plans

Despite the current scenario, it is unjust to mention that Nepal government has not given a thought on this issue; the outcomes though are quite futile. It is found that Nepal government has included environment quality in its plan and strategy and formulated national policies and legislations on this regard beginning from the middle of the 1990s. The important national plans and legislations of Nepal government on environmental issue can be listed as follows [40]:

- Environmental policy and legislative framework: Environmental Act 1996 and Regulation 1997, National Climate Change Policy 2011, National Low Carbon Economic Development Strategy (still in draft), and National Pollution Control Strategy and Action Plan (still in draft)
- Transport Sector Policies and Legislations: National Transport Policy 2001, Transport Management Act 2049 (Nepalese calendar year), Vehicles and Transport Management Rules 2054 (Nepalese calendar year), and National Sustainable Transport Strategy (NSTS) (2015-2040) (still in draft)
- Industry Sector Policies and legislations: Industrial Policy 2011, Foreign Direct Investment Policy 2015, and Industrial Enterprises Act 2073 (Nepalese calendar year)
- Energy Sector Policies and Legislations: Hydropower Development Policy 2001, Rural Energy Policy 2006, and Renewable Energy Subsidy Policy 2016.

Besides formulating the abovementioned policies and legislatives, Nepal Government has also formed various committees to deal with the air pollution problem, as mentioned below:

- Task Force on Air Pollution Control in Kathmandu Valley, 2073 (Nepalese calendar year)
- High Level Committee on Probing and Solving the Issues on 20 year Old Vehicles, 2058 (Nepalese calendar year)
- Committee on Implementation of the Order of Supreme Court on Phase out of 20 Year Old Vehicles, 2058/59 (Nepalese calendar year)
- Committee on Review of Vehicle Emission Standard and Monitoring Mechanism 2060. (Nepalese calendar year)
- Technical Committee on the Relocation of Brick Industries from Kathmandu Valley 2060 (Nepalese calendar year).

Nepal government claims to be seeking to make use of a rich Air Quality Management portfolio created by big cities of developed and developing countries, for implementing and executing successful programs while avoiding many of the recognized pitfalls. Some of the future prospects include the following:

- Strategies on Ambient Urban Air Quality Management: with the vision that all the citizen living or visiting urban cities of Nepal breathe clean air
- Action Program on Ambient Air Quality Management of Kathmandu Valley: the aim is to bring the level of air pollution in the valley to the target set in the National Ambient Air Quality Standard of Nepal within the next 5 years. The various factors to support this plan are Air Quality Management supporting system, Environmentally Sustainable Transport System, Environment Friendly Construction Activities, Reducing Emissions of Industries in Valley, Environmentally Sound Management of Wastes (dealing with toxic air pollutants), Promoting Cleaner Fuel and Technology to Minimize Domestic Pollution (Indoor Air Pollution), Strengthening the Policy and Legislative Framework, Institutional Arrangement for Effective Implementation, and Financing the Action Plan.

## 6. Areas to Be Addressed

Nepal government has been formulating policies to control environmental pollution since the 1990s, but the implementation of the legislatives has not been effective enough. Several seminars, talks, committees, and task forces have been formulated to curb the issue but no concrete solution has been met. Air pollution has been a burning issue but adequate air quality monitoring stations have been limited only to a few places like Kathmandu, Kavre, Pokhara, Chitwan, and Rupendehi (http://pollution.gov.np). No sufficient study regarding air quality of urban and suburban regions has been done and categorically published on the basis of pollution level. It has been generalized that poor air quality is having adverse effect on people's health; however, studies are limited and no sufficient studies have been done longitudinally to find out short and long term effects, seasonal patterns, geographical variations, and other issues of air quality affecting human health. Knowledge and awareness of poor air quality's threat on human health have not reached the common public level which has blind folded them from taking basic precaution measures. Besides, other hindrances are economic conditions, malpractice in politics, and limited approach to health facilities.

## 7. Conclusion

Air pollution has been a huge burden to the residents of Kathmandu, threatening the lives of thousands of people of every year. The scenario is obvious to worsen in the coming years if immediate preventive measures are not taken in time. It is of utmost urgency to educate the common people on harmful aspects of air pollution and the necessary precautions to prevent its deadly consequences. The solution to Kathmandu's air pollution can be achieved only when the government takes the leading role in addressing the situation. The Constitution of Nepal 2015 has mentioned that clean and healthy environment should be guaranteed to the people as their primary right [58]. National health policy of Nepal has included air pollution as a priority research/public health agenda, but implementation part has not been efficient. Benefit of doubt can be given to government as the political scenario is still in the transition phase after the Nepalese overthrew centuries-old monarchy and established the Federal Democratic Republic of Nepal. Currently, people are awaiting a better political stability whereby a better economic growth can be achieved so that solutions to this public health issue be achieved.

## Abbreviations

NCD: Noncommunicable Disease
WHO: World Health Organization
CO: Carbon monoxide
NO2: Nitrogen dioxide
SO2: Sulfur dioxide
EPI: Environmental Performance Index
PM: Particulate Matter
COPD: Chronic obstructive pulmonary disease
IHD: Ischemic Heart Disease
ALRTI: Acute Lower Respiratory Tract Infection
DoH: Department of Health
GDP: Gross Domestic Product
TSP: Total Suspended Particles
PM: Particulate Matter
O3: Ozone
ARI: Acute respiratory infection.
